# Analysis of the Combined Effects of a Novel Combination of Hypersmin, Pumpkin Seed and Amaranthus Extracts in an In Vitro Model of Chronic Venous Insufficiency

**DOI:** 10.3390/nu17111807

**Published:** 2025-05-26

**Authors:** Sara Ferrari, Rebecca Galla, Simone Mulè, Claudio Molinari, Francesca Uberti

**Affiliations:** 1 Laboratory of Physiology, Department for Sustainable Development and Ecological Transition, 13100 Vercelli, Italy; 2 Noivita Srls, Spin Off University of Piemonte Orientale, Strada Privata Curti 7, 28100 Novara, Italy

**Keywords:** natural extracts, oral supplementation, 3D in vitro vein model, varicose veins, vein thrombosis

## Abstract

**Background**: Venous hypertension is the primary cause of the disorder known as chronic venous insufficiency (CVI), which affects the lower extremities’ venous system. Because of its biological proper ties, which include anti-inflammatory, antioxidant, and vascular tone enhancement, medicinal herbs and natural substances are highly recommended for treating CVI. Therefore, this study examined the advantages of a novel combination composed of hypersmin, pumpkin seed and amaranthus extracts (named MIX) in modulating different parameters involved with CVI. **Methods**: The capacity of these natural compounds to pass across the intestinal barrier and reach the bloodstream was examined using a 3D intestinal barrier model that mimics oral ingestion. The biological effects of the MIX were then compared to those of a commercial product using an in vitro CVI model. **Results**: The findings demonstrate that the new MIX significantly reduced inflammation while increasing nitric oxide production. The MIX was more successful than the commercial product in reducing apoptosis while restoring vasal tone and extracellular matrix activity. **Conclusions**: This work has therefore demonstrated the positive benefits of extracts from amaranthus, pumpkin seed and hypersmin in the context of CVI, raising the prospect of creating a unique combination for patients with CVI.

## 1. Introduction

Chronic venous insufficiency (CVI) is a global condition characterised by a range of clinical signs and symptoms, from asymptomatic varicose veins and spider veins to venous leg ulcers. It is generally defined by reduced venous blood flow, which results in venous hypertension [1]. This condition leads to pathological changes such as lower extremity swelling, skin trophic abnormalities, and pain. If untreated, CVI can progress to post-thrombotic syndrome and venous ulcers, with symptoms including discomfort, leg swelling, pruritus, skin discoloration, limb heaviness, and swelling that improves with elevation [2]. CVI is more than a cosmetic issue; it increases the risk of venous thrombotic events and is associated with significant impairments in daily activities and quality of life [3]. A complex interplay of environmental factors, such as jobs involving heavy lifting or prolonged standing, genetic predisposition, and age-related loss of structural integrity in the leg venous system contribute to this condition [4]. Additionally, older age, female sex, obesity, pregnancy, and a history of deep vein thrombosis are recognised risk factors. However, it remains unclear which specific variables contribute to the development of CVI, making the identification of at-risk individuals challenging [5].

It is known that venous disease occurs when venous pressure rises, coupled with several processes that hinder blood flow. Indeed, venous hypertension can result from venous reflux, a condition characterised by blood backflow due to malfunctioning valves. The persistence of this condition disrupts the reduction–oxidation balance [6]. Structural changes such as venous obstruction and valvular reflux [7], or functional changes can lead to venous hypertension [4]. Furthermore, inflammation in venous circulation, which results in increased inflammation of the vein wall and the extravasation of inflammatory cells and molecules into the interstitium, is a significant factor associated with CVI [8]. Indeed, an inflammatory response to venous incompetence is believed to be the source of pain in CVI. This response is triggered by venous stasis, which causes endothelial cells to release growth factors and inflammatory mediators, leading to the characteristic pain of CVI [9]. In correlation with venous inflammation, there is also an overproduction of reactive oxygen species (ROS) that drives persistent local inflammation, resulting in an imbalance in the antioxidant system [10,11]. Indeed, injured veins have been shown to exhibit reduced antioxidant potential, while simultaneously presenting higher levels of nitric oxide (NO) [12]. Evidence suggests that NO may be involved in endothelial proliferation and increased vascular permeability, as well as in the maintenance of the function of the microcirculatory endothelial barrier [13]. Due to a lack of scientific knowledge regarding the disease’s aetiology, there are currently no approved treatments to stop or reduce its growth. However, considering that natural substances such as flavonoids have antioxidant properties and drive endothelial cells to release NO, the research is focusing on natural substances to manage CVI. Diosmin is one flavonoid used to treat varicose veins as it enhances lymphatic activity, microcirculatory flow, microvascular permeability and venous tone [14]. Diosmin is currently one of the most sought-after natural substances in the treatment of CVI because of its positive effects on the cardiovascular system. According to clinical studies, diosmin improves lymphatic drainage and microcirculation, reducing oedema [15]. In addition, it decreases capillary filtration and hyperpermeability while raising capillary resistance, vascular tone and vein flexibility. By blocking proinflammatory cytokines via NFκB pathways, diosmin also has an anti-inflammatory impact [16]. Generally, treatment with diosmin improves the quality of life for CVI patients, and symptoms, including pain, oedema and tightness, are reduced. Indeed, by lowering ROS produced during inflammation, which can harm tissue and release inflammatory mediators, diosmin’s antioxidant activity may also lessen the symptoms of CVI [17]. These beneficial effects explain why diosmin is already used in commercial products to reduce the symptoms of CVI. Hesperidin, another chemical formed from citrus fruits like diosmin, is typically combined with diosmin to maximise its positive benefits. Much research has demonstrated that this combination improves microcirculatory performance and reduces venous inflammation. Additionally, studies have shown that it can improve quality of life while lowering oedema and pain [18]. To date, there is growing evidence that the combination of diosmin and hesperidin has a positive effect on the pathophysiological processes underlying chronic venous disease, particularly with regard to microcirculation [19]. However, the use of this combination is not sufficient to the management of CVI condition. For this reason, it would be interesting to add other natural extracts with antioxidant action to this combination.

For example, pumpkin is already well-known for its positive effects on the cardiovascular system owing to its ability to lower cholesterol levels, and for containing numerous phytosterols that help reduce inflammation in the body [20]. Another significant component of pumpkin is vitamin E, which is present in substantial amounts and is recognised for enhancing skin tone and promoting faster skin healing and regeneration [21]. Specifically regarding CVI, it has been demonstrated that pumpkin oil provides firmness and flexibility to the blood vessel walls, reduces swelling and inflammation and alleviates the sense of heaviness and fatigue in the legs [22]. The ability of pumpkin to exert these positive effects can be attributed to content of flavonoids, organic compounds that regulate the functioning of the circulatory system, are essential for blood flow and seem protective for the treatment of vascular disorders [13]. These attributes make pumpkin a strong candidate for developing novel therapies for CVI.

Amaranthus is an additional compound that may assist in developing novel treatments for CVI. Due to its rutin content, amaranthus enhances the circulatory system, tissues and capillaries, while also counteracting the formation of varicose veins [23]. Indeed, by inhibiting thromboxane A2 and platelet-activating factor (PAF), rutin’s action against free radicals can help preserve the integrity of these walls and decrease capillary permeability [24]. The antioxidant, antihypertensive and antithrombotic properties of amaranthus are attributed to various encrypted amino acid sequences [25], making it a beneficial extract for use in the context of CVI.

Thus, after collecting evidence about different natural substances that could be useful in managing CVI, this study aimed to develop a novel oral formulation containing diosmin, hesperidin (called hypersmin), pumpkin and amaranthus that could mitigate CVI after intestinal absorption.

## 2. Materials and Methods

### 2.1. Agent Preparation

Research was conducted to ascertain whether the substances under examination (amaranthus, pumpkin seeds and hypersmin) may penetrate the intestinal barrier and have advantageous effects at the venous level. All the compounds were acquired from NutraFutura srls (Legnano, MI, Italy). Table 1 shows the characterisation of the extracts.

These compounds were tested separately and in combination and were validated by dose–response examinations, which showed that amaranthus 50 mg, pumpkin seeds 250 mg and hypersmin 289 mg were the most effective concentrations. Dulbecco’s Modified Eagle’s Medium (DMEM), acquired from Merck Life Science in Rome, Italy, was used to prepare each tested material. It contained no phenol red and was supplemented with 1% penicillin–streptomycin, 0.5% foetal bovine serum (FBS) and 2 mM L-glutamine (all from Merck Life Science, Rome, Italy). All the analyses were performed and compared to a commercial product. The commercial product used for comparison, consists mainly of 500 mg of bioflavonoids (450 mg Diosmin and 50 mg Hesperidin). Substances were diluted 1:2000 to replicate the human dose in vitro [26].

### 2.2. Cell Cultures

Derived from human Caucasian colon adenocarcinoma, the Caco-2 cell line was acquired from the American Type Culture Collection (ATCC, Manassas, VA, USA). It was cultivated in DMEM Advance (DMEM-Adv, Thermo Fisher Scientific, Rodano, MI, Italy) supplemented with 10% FBS (Merck Life Science, Rome, Italy), 2 mM L-glutamine and 1% penicillin–streptomycin (all from Merck Life Science, Rome, Italy). The cells were kept at 37 °C in an incubator with 5% CO_2_. The Food and Drug Delivery (FDA) and the European Medicines Agency (EMA) have approved the use of this cell line in experimental models that forecast drug absorption, metabolism and bioavailability after oral delivery [27,28,29]. 1 × 10^4^ cells were plated in 96-well plates and subjected to analysis using an MTT-based In Vitro Toxicology Assay Kit (MTT, Merck Life Science, Rome, Italy) to determine the viability of the cells. To conduct an absorption investigation, 2 × 10^4^ cells were plated in a 24-well plate using 6.5 mm Transwell^®^ inserts and a polycarbonate membrane with 0.4 μm pores. The cells were cultured for 8 h in an incubator using DMEM-Adv media free of red phenol before stimulation. 1% penicillin–streptomycin, two mM L-glutamine and 0.5% foetal bovine serum were added to the medium [28]. The ATCC provided the human umbilical vein endothelial cell line, HUVEC, cultivated in a flask coated with Endothelial Growth Medium-2 (EGM-2) and 0.1% gelatin. Supplements to EGM-2 (Lonza, Basel, Switzerland) included the following components: 0.1% recombinant analogue of human insulin-like growth factor-I, 0.4% VEGF, 0.4% human basic fibroblast growth factor, 0.1% ascorbic acid, 0.1% human epidermal growth factor, 0.1% gentamicin sulphate–amphotericin and 0.1% heparin [30]. To create an in vitro model of veins, 1 × 10^5^ HUVEC cells/cm^2^ were plated on the basal compartment of the Transwell^®^ on a Matrigel layer (BD Biosciences, Meylan, France) and stimulated with the basolateral medium of Caco-2 cells to investigate cell viability analysis and ROS production analysis and the mechanisms involved in venous insufficiency. The cells placed were treated with KCl to mimic venous insufficiency [13].

### 2.3. Experimental Protocol

This study was subdivided into two steps: intestinal and venous analyses. In the first phase, a 3D in vitro model of the intestinal barrier, approved by EMA and FDA [31,32], was treated with amaranthus, pumpkin seeds and hypersmin, and their combination for a period ranging from 1 to 6 h. After the treatment period, the absorption mechanisms of individual agents, their combination and a commercial product were analysed. After that, in the second phase, the metabolisation of intestinal cells was used to stimulate HUVEC cells placed in the basolateral compartment on a Matrigel layer to recreate a vein model in vitro. The venous model was treated with all test substances every 12 h in a timeframe from 12 to 48 h, and at the end of every period, the cells were damaged with 96 mM KCl for 15 min [13]. These cells’ viability and oxidative/inflammatory status were analysed each time. Further, the mechanisms of maintaining vessel tone were evaluated by assessing endothelin-1, eNOS and NO production. The apoptotic pathway was also investigated by analysing BAX, Cyto-C and Caspase 3. Finally, some markers involved in maintaining vessel architecture were also examined, specifically two metalloproteases (MMP) such as MMP-3 and MMP-9.

### 2.4. In Vitro Model of Intestinal Barrier

The FDA and EMA approved a standard approach published in the literature to estimate the absorption, metabolism, and bioavailability of various treatments after oral intake by humans [31,32]. These methods used the Transwell^®^ technology to replicate the intestinal barrier model in vitro. After 21 days of cultivation in a complete media before the simulations [33], the trans-epithelial electrical resistance (TEER) of CaCo-2 cells was measured using EVOM3 with STX2 chopstick electrodes (World Precision Instruments, Sarasota, FL, USA). As demonstrated by TEER values of 400 Ωcm^2^, this monitoring enabled the assessment of mature intestinal epithelial development and the emergence of a suitable paracellular mechanism around day 21. Before stimulation, the medium on the apical side was brought to the small intestine’s lumen pH of 6.5. The pH was 7.4 on the basolateral side, indicating blood [33]. At each defined time period, intestinal absorption or bioavailability was assessed by using 0.04% fluorescein (Merck Life Science, Milan, Italy), a fluorescent marker dye was used to determine transepithelial transit, as it is a fluorescent contrast agent that is inert, safe and has been used in many clinical studies [34]. The amount of fluorescein transported was measured at 37 °C for 40 min by incubating Caco-2 cells at the above concentration. Fluorescence was detected with a fluorescence spectrophotometer (Infinite 200 Pro MPlex, Tecan, Männedorf, Switzerland) at excitation/emission wavelengths of 490/514 nm. The following formula was used to convert the acquired data into absorption:

Papp = dQ/dt ⇥ 1/m0 ⇥ 1/A ⇥ V Donor,

where

dQ: denotes the amount of material being carried, expressed in nanomoles (nmol) or micrograms (μg);

dt: denotes the incubation period’s duration, expressed in seconds (s);

m0: denotes the initial quantity of substrate applied to the donor compartment, expressed in micrograms (μg) or nanomoles (nmol);

A: The Transwell^®^ membrane’s external area is measured in cm^2^;

V Donor: the volume of liquid (in cm^3^) in the donor compartment.

In order to rule out the impact of Transwell membranes, negative controls without cells were examined.

### 2.5. MTT Cell Viability Assay

All cell types were subjected to the MTT-based In Vitro Toxicology Assay Kit (Merck Life Science, Rome, Italy), per the literature [35]. Following stimulation, the cells were incubated for two hours at 37 °C with 1% MTT dye. The purple formazan crystals were dissolved using comparable amounts of MTT Solubilization Solution. Cell viability was assessed by measuring absorbance at 570 nm and correction at 690 nm (Infinite 200 Pro MPlex, Tecan, Mannedorf, Switzerland). The results were compared to the control (untreated samples, represented by the 0% line) and were shown as the means (%) SD of five distinct tests conducted in triplicate.

### 2.6. ROS Production Assay

Cytochrome C reduction in HUVEC supernatants was measured at 550 nm using a standard methodology [36] to quantify superoxide anion release (Infinite 200 Pro MPlex, Tecan, Männedorf, Switzerland). Compared to the control value (0 line), the O_2_ rate was expressed as the mean SD (%) of nanomoles per reduced cytochrome C per microgram of protein.

### 2.7. Tumour Necrosis Factor α (TNFα) ELISA Kit

Following the standard procedure, the TNFα ELISA kit (Merck Life Science, Rome, Italy) was used to measure the quantity of TNFα in HUVEC cells [37]. Using a spectrophotometer (Infinite 200 Pro-MPlex, Tecan, Männedorf, Switzerland), the colorimetric intensity was measured at 450 nm. By creating a calibration curve between 24.58 pg/mL and 6000 pg/mL, the findings were computed and presented as a percentage concerning the control of five independent tests carried out in duplicate.

### 2.8. Interleukin 1β (IL-1β) ELISA Kit

Following the manufacturer’s instructions, the lysates of HUVEC cells were examined using an IL-1β ELISA kit (R&D Systems, Minneapolis, MN, USA). A spectrophotometer (Infinite 200 Pro MPlex, Tecan, Männedorf, Switzerland) was used to read the plate at 450 nm with correction at 570 nm. The OD of the samples was compared to a standard curve, which ranged from 3.906 to 250 pg/mL. Five separate experiments were conducted in duplicate, and the findings were presented as mean ± SD (%) vs. control (0% line).

### 2.9. Analysis of NO Production

Following the manufacturer’s instructions, a kit (Griess assay, Promega, Milano, Italy) was utilised to measure the generation of NO in the HUVEC supernatant following stimulation [38]. A spectrometer (Infinite 200 Pro-MPlex, Tecan, Männedorf, Switzerland) measured the samples’ absorbance at 520–550 nm wavelengths. Based on the standard curve generated using standard nitrate, the results were expressed as a percentage (%) normalised to the untreated samples. Five separate tests were conducted in duplicate, and the findings were presented as mean ± SD (%) compared to control (line 0).

### 2.10. eNOS ELISA Kit

The eNOS concentration was measured following the manufacturer’s instructions for the DuoSet ELISA kit for Human eNOS (R&D Systems, Minneapolis, MN, USA) in HUVEC cells [39]. Using a spectrometer (Infinite 200 Pro-MPlex, Tecan, Männedorf, Switzerland), the absorbance of the samples was measured at 450 nm. The results were displayed as a percentage (%) normalised to the untreated samples about the standard curve (78.1–5000 pg/mL) created using a standard. After five tests were run in triplicate, the results were shown as mean ± SD (%) vs. control (line 0).

### 2.11. Endothelin-1 ELISA Kit

According to the manufacturer’s instructions, the Human Endothelin 1 ELISA Kit (MyBiosource, San Diego, CA, USA) measured endothelin-1 in HUVEC cells [40]. A spectrometer (Infinite 200 Pro MPlex, Tecan, Männedorf, Switzerland) quantified the absorbance at 450 nm. Regarding the standard curve (31.25–2000 pg/mL), the results of five separate tests conducted in triplicate were presented as a percentage (%) normalised to the untreated samples (line 0).

### 2.12. Apoptosis Analysis

Human Bax ELISA Kit (MyBiosource, San Diego, CA, USA), Cytochrome-C ELISA Kit (MyBiosource, San Diego, CA, USA), and Human Caspase 3 (Cleaved) ELISA Kit (ThermoFisher, Waltham, MA, USA) were employed, following the manufacturer’s guidelines, to analyse the levels of Bax, Cytochrome-C, and Caspase 3 in HUVEC lysates. The absorbance at 450 nm was measured using a spectrophotometer (Infinite 200 Pro MPlex, Tecan, Männedorf, Switzerland). The standard curve ranges from 0 to 2000 pg/mL for Bax, 15.6 to 500 nmol/L for cytochrome-c and 0.039 to 2.5 ng/mL for caspase-3, about the data. The results of five experiments were presented in triplicate as a percentage (%) compared to the control (0 line).

### 2.13. MMPs Analysis

The Human Total MMP-3 ELISA Kit-Quantikine (R&D Systems, Minneapolis, MN, USA) and the Human MMP-9 ELISA Kit-Quantikine (R&D Systems, Minneapolis, MN, USA) were utilised following the manufacturer’s instructions [41,42] to analyse the levels of MMP3 and MMP9 in HUVEC cells. Absorbance at 450 nm was measured using a spectrophotometer (Infinite 200 Pro MPlex, Tecan, Männedorf, Switzerland). Compared to the data, the standard curve spans from 0.2 to 10 ng/mL for MMP3 and from 0.3 to 20 ng/mL for MMP9. The results of five different experiments were then presented in triplicate as a percentage (%) relative to the control (0 line).

### 2.14. Statistical Analysis

For each experimental technique, the data were presented as the mean ± SD of at least five biological replicates, each conducted three times. Using GraphPad Prism 10.2.3 (GraphPad Software, La Jolla, CA, USA), we employed one-way ANOVA with either Mann–Whitney’s U test or Bonferroni’s post hoc test, as applicable, to perform statistical comparisons across groups. The threshold for statistical significance was set at *p* < 0.05. The data from all other experimental protocols were normalised to the control values set at 0%. 

## 3. Results

### 3.1. Absorption Rate Analysis in an In Vitro Intestinal Barrier Model

**Figure 1:**
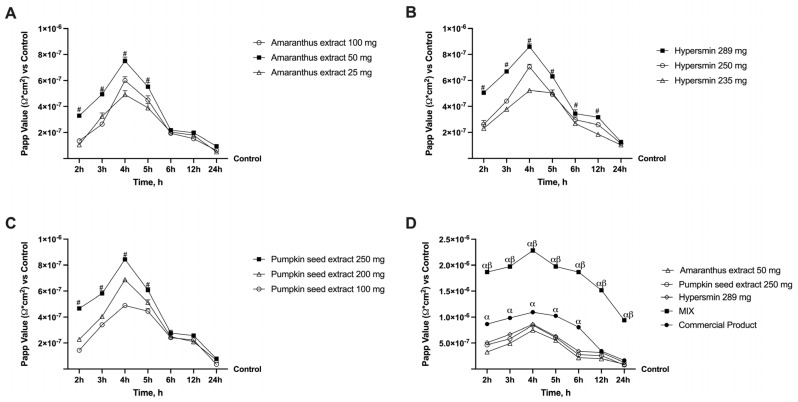
Screening on in vitro model of the intestinal barrier. In (**A**) the Permeability values of Amaranthus extract; in (**B**) the Permeability values of Hypersmin; in (**C**) the Permeability values of Pumpkin seed; in (**D**) the Permeability values of MIX in comparison with a commercial product; data < 0.2 × 10^−6^ cm/s mean very poor absorption with a bioavailability < 1%, data between 0.2 × 10^−6^ and 2 × 10^−6^ cm/s with bioavailability between 1 and 90%, and data > 2 × 10^−6^ cm/s mean excellent absorption with a bioavailability over 90%. The data are reported as means ± SD (%) of five independent experiments performed in triplicate and normalised to control values (the 0% line). From (**A**) to (**C**), all the agents *p* < 0.05 vs. Control; # *p* < 0.05 vs. other concentrations. In (**D**), all the agents *p* < 0.05 vs. Control; α *p* < 0.05 vs. single agents; β *p* < 0.05 vs. commercial product.

An initial set of experiments was conducted on different concentrations of amaranthus, hypersmin and pumpkin seed to evaluate the dosage that gave the best results regarding absorption. These analyses were performed using 0.04% fluorescein, a probe is commonly used to assess permeability. In Figure 1, all the absorption data at different concentrations have been reported. The data showed that all dosages of amaranthus possess a higher absorption rate than the control (*p* < 0.05); among them, amaranthus 50 mg demonstrated a significantly higher value from 2 h to 5 h compared to amaranthus 100 mg and amaranthus 25 mg (Figure 1A, *p* < 0.0001). Even from 6 h to 24 h, amaranthus 50 mg maintained a higher absorption profile than the other two concentrations tested, but not significantly. Nevertheless, amaranthus 50 mg had the best absorption profile, so this concentration was chosen for the following experiments. All the hypersmin concentrations tested also exerted a significant increase in the absorption rate compared to the control (Figure 1B, *p* < 0.05); specifically, in this case, a higher result was obtained with the higher concentration tested (289 mg). Indeed, hypersmin 289 mg maintained a higher absorption profile from 2 h to 12 h compared to hypersmin 250 mg and hypersmin 235 mg (*p* < 0.01). At 24 h, the absorption profile of the concentration chosen is quite similar. According to the data collected, hypersmin 289 mg was selected for the subsequent experiments. Finally, all pumpkin seed concentrations tested also demonstrated an increase in the absorption rate compared to the control (Figure 1C, *p* < 0.05). Pumpkin seed extract 250 mg obtained the highest value regarding absorption, with a significance over other two concentrations analysed from 2 h to 5 h (*p* < 0.0001). As was observed for amaranthus, in this case, from 6 h to 24 h the absorption profile of the different concentrations is also similar between them; however, pumpkin seed extract 250 mg remains the best absorbed, which is why this concentration was chosen. After selecting the singular concentrations, the extracts under examination were analysed together. This MIX was then compared to a commercial product to verify if this novel formula could produce better beneficial results than a product already on the market. As shown in Figure 1D, the MIX was characterised by the best absorption profile for all the time points analysed, compared to the single agents and the commercial product (*p* < 0.0001). Specifically, the MIX reached the highest absorption values at 4 h with an increase of about 1 time, 2 times, 1.6 times and 1.7 times compared to the commercial product, amaranthus extract, hypersmin and pumpkin seed extract, respectively (*p* < 0.0001). Thus, these data demonstrated that a great absorption profile characterises the MIX.

### 3.2. Effect of Single Agents and Their Combination on a 3D Vein Model

**Figure 2:**
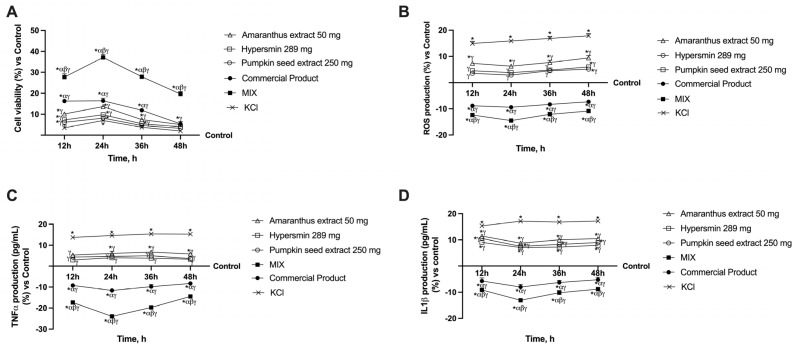
Safety and anti-inflammatory analysis on in vitro model of vein after intestinal passage. In (**A**) cell viability through MTT test; in (**B**) ROS production through the analysis of cytochrome C reduction; in (**C**) TNFα production with specific ELISA kit; in (**D**) IL-1β production with specific ELISA kit; * *p* < 0.05 vs. Control; α *p* < 0.05 vs. single agents; β *p* < 0.05 vs. commercial product; γ *p* < 0.05 vs. KCl.

To understand the effects of the single agents, MIX and the commercial product on an in vitro vein model, a gut–vein axis was created in vitro to analyse the effects of all the compounds after intestinal passage. These analyses were performed from 12 h to 48 h to mimic a double administration of the compounds throughout the day and evaluate their effects over time; further, all the analyses on the vein model were performed in a damaged condition induced with 96 mM KCl. The first test evaluated the compound’s effect after intestinal absorption on cell viability. As can be seen in Figure 2A, from 12 h to 36 h all the compounds exerted an increase compared to the control (*p* < 0.001); similar data were obtained for the comparison to KCl (*p* < 0.05), however, at 36 h the values obtained after pumpkin seed extract treatment are not significant compared to KCl. When comparing the two formulations to the individual agents, both are significant for the latter from 12 to 36 h. Still, only the MIX is substantial for the individual agents at 48 h (*p* < 0.05, about four times more than the single agents).

Furthermore, the MIX always has a significant effect on the commercial product (*p* < 0.0001). Based on these findings, it can be concluded that all of the studied agents positively affect venous cells and that the MIX produces the best results when venous insufficiency-like circumstances are present (*p* < 0.0001). After that, ROS production was analysed (Figure 2B) as ROS have a key role in endothelial damage and the development of CVI. Indeed, KCl (used to mimic CVI) induced a higher production of ROS compared to all the other agents under examination (*p* < 0.0001). In contrast, the individual agents were able to limit this production by reducing ROS production levels to levels that were only marginally higher than the control in the case of amaranthus (*p* < 0.05) and comparable to the control in the cases of hypersmin and pumpkin seed extract. These promising findings are amplified when the two formulations are compared; indeed, both reduce KCl-induced production and ROS generation to levels lower than the individual agents and notably lower than the control (*p* < 0.0001). In detail, the MIX outperforms all other agents with the lowest and most significant amounts of ROS production (*p* < 0.0001, about 54% more than commercial product at 24 h). TNFα and IL-1β production were also analysed (Figure 2C,D) as persistent inflammation characterises chronic venous disease. After treatment with KCl alone, their production was significantly increased for both the parameters examined compared to the control (untreated cells, *p* < 0.0001). Hypersmin and pumpkin seed extract caused a consistently considerable drop in TNFα production compared to KCl, bringing the values back to the control level. Compared to KCl, amaranthus also consistently reduced the production of TNFα (*p* < 0.0001), albeit the results were slightly more significant than the control. Further, TNFα production was consistently reduced by the MIX and the commercial product compared to KCl, the individual treatments, and the control (*p* < 0.0001). If only the MIX is considered, these data are considerably more favourable because the latter is always significant compared to the commercial product (*p* < 0.0001, about 1 time more than commercial product at 24 h), suggesting a more potent anti-inflammatory effect. Analyses of IL-1β production yielded similar results, although, in this instance, none of the individual agents could restore the levels to the control one (untreated cells); notwithstanding, the production was still lower than that of KCl (*p* < 0.0001). Again, the MIX and the commercial product induce a substantial decrease in levels compared to KCl, the single treatments and especially the control (*p* < 0.0001). Additionally, the MIX exerts the most significant effect against the commercial product (*p* < 0.0001), thus confirming the data on a more significant anti-inflammatory effect.

**Figure 3:**
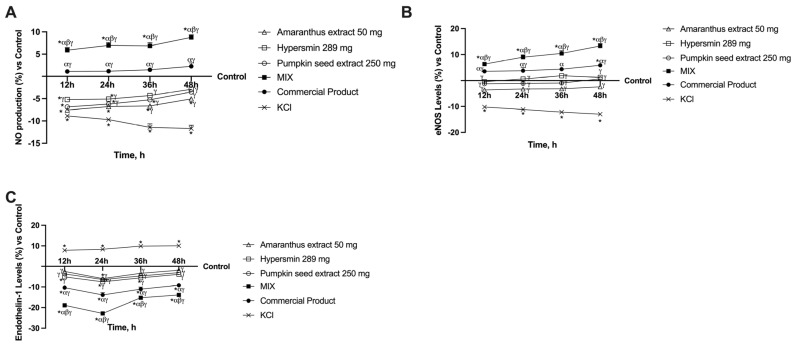
Vascular tone analysis on in vitro model of vein after intestinal passage. In (**A**) NO production; in (**B**) eNOS levels; in (**C**) endothelin-1 levels. From (**A**) to (**C**) all the data are obtained with specific ELISA kits; * *p* < 0.05 vs. Control; α *p* < 0.05 vs. single agents; β *p* < 0.05 vs. Commercial product; γ *p* < 0.05 vs. KCl.

The synthesis of NO and the levels of its enzyme eNOS were examined since NO has a variety of functions in the vasculature, including preserving vascular tone and averting endothelial dysfunction. NO production analysis (Figure 3A) revealed that from 36 h onward, all the single agents can drastically lower its production compared to the damaged state (*p* < 0.0001). In contrast, the formulations provided the same effect after 12 h. More specifically, the commercial product had a moderately higher effect than the control, whereas the MIX induced NO production significantly higher than the control at all time points with a peak of about 10% at 48 h (*p* < 0.0001). This parameter’s analysis also showed that the MIX has a more substantial effect than the commercial product (*p* < 0.0001, about three times more at 48 h). The levels of the enzyme responsible for NO production were then analysed. eNOS analysis (Figure 3B) revealed that the single agents induced an increase in the levels compared to KCl (*p* < 0.0001) and similar to the control (untreated cells) at all time points analysed, and hypersmin induced the highest effect at 36 h; indeed, at this time, the commercial product was not able to cause a significantly greater effect compared to hypersmin. However, at the other timeframe, the commercial product induces a more significant effect than the single agents (*p* < 0.05). Regarding the MIX, it induced a significant effect during the timeframe compared to KCl, single agents, control and commercial product (*p* < 0.0001, about 1.3 times more than the commercial product at 48 h), thus demonstrating its ability to influence the synthesis of NO in a superior manner. Endothelin-1 is another element that affects vascular tone and is elevated in CVI patients. These data were also confirmed by the analysis of this parameter following treatment with KCl, which induced a significant increase in endothelin-1 levels compared to the untreated cells (*p* < 0.05, Figure 3C). All the single compounds alone decreased endothelin-1 levels compared to KCl (*p* < 0.001). Further, both formulations decreased the levels of endothelin-1 for all timeframes analysed (*p* < 0.05 vs. single agents and KCl). In detail, the effect of the MIX remains significant compared to the control and to the commercial product (*p* < 0.0001, about 60% more than commercial product at 24 h), with a peak at 24 h and maintenance of stable levels at 36 h and 48 h. It seems evident from the data collected thus far that the MIX controls vessel tone more effectively than the commercial product.

### 3.3. Effects of Single Agents and Their Combination on Apoptosis in CVI Condition

**Figure 4:**
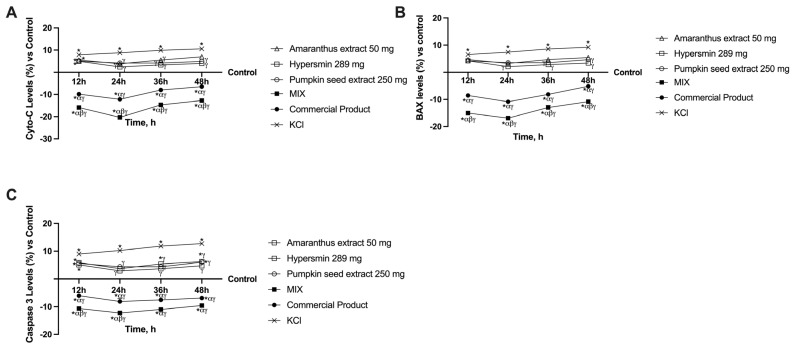
Apoptosis pathway analysis on in vitro model of vein after intestinal passage. In (**A**) Cyto-C levels; in (**B**) BAX levels; in (**C**) Caspase-3 levels. From (**A**) to (**C**) all the data are obtained with specific ELISA kits; * *p* < 0.05 vs. Control; α *p* < 0.05 vs. single agents; β *p* < 0.05 vs. Commercial product; γ *p* < 0.05 vs. KCl.

Different studies have demonstrated that apoptotic deregulation is present in primary varicose veins; precisely, apoptotic markers such as Cyto-C, BAX and Caspase 3 were increased in patients suffering from primary varicose veins [43]. Indeed, all these markers were increased by the treatment with KCl 36 mM (*p* < 0.0001 vs. control). Regarding Cyto-C levels (Figure 4A), all the single agents could reduce the damage caused by KCl starting from 24 h (*p* < 0.001). In contrast, the formulations decreased Cyto-C levels compared to KCl, the single agents, and the control at all the timepoints analysed (*p* < 0.0001). Although the two formulas in this instance have a comparable trend over time, peaking at 24 h, the MIX consistently produces the more substantial effect (*p* < 0.0001, about 66% more at 24 h vs. the commercial product). Concerning BAX analysis (Figure 4B), amaranthus could not significantly decrease KCl effects over time. Hypersmin could reduce this effect from 24 h and pumpkin seed extract from 36 h (*p* < 0.05 vs. KCl). Once again, the commercial product and the MIX induced a decrease at all time points compared to KCl, the single agents, and the control (*p* < 0.0001). Further, the most potent effects of the MIX compared to the commercial product were confirmed (*p* < 0.0001, about 54% more at 24 h vs. the commercial product). Finally, Caspase 3 levels were analysed to provide a complete vision of the apoptotic pathway (Figure 4C). In this case, starting from 24 h, all the single agents were able to reduce Caspase 3 levels compared to KCl (*p* < 0.001). Further, the data obtained are comparable to that of the untreated cells (control) for all the agents at 24 h, for amaranthus and pumpkin seed extracts at 48 h and only for hypersmin at 72 h. Again, the commercial product and the MIX induced a decrease compared to KCl, the single agents and the control (*p* < 0.0001). This time, however, the comparison of the two formulations produced different results from previous analyses: while the MIX always yields a higher increase than the commercial product, these data are only significant at 12 h and 24 h (*p* < 0.001, about 51% more at 24 h vs. the commercial product), whereas at 36 h and 48 h, the difference between the two becomes less noticeable. Regardless, when taken together, these findings show the potent anti-apoptotic action of the novel MIX under study compared to the commercial product.

### 3.4. Ability of Single Agents and Their Combination to Decrease MMPs Levels in CVI Condition

**Figure 5:**
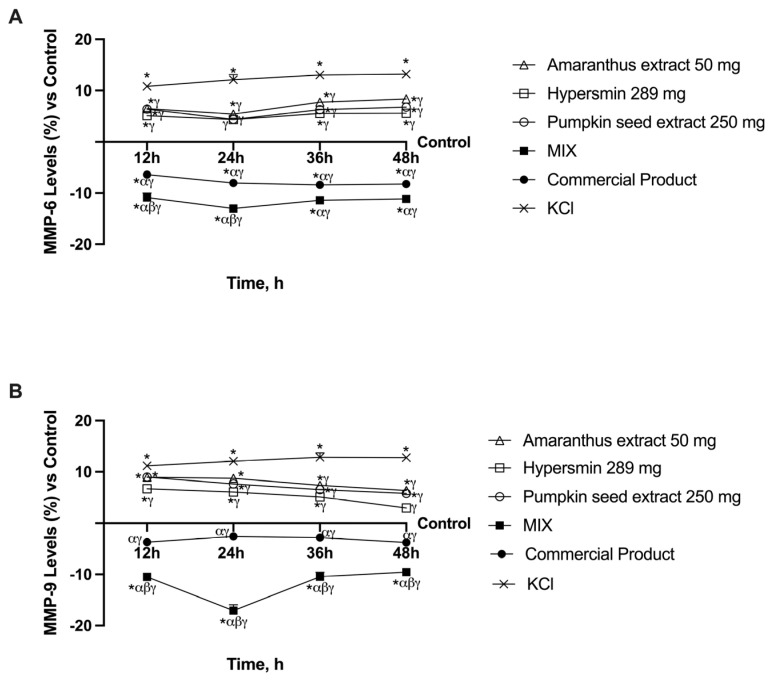
Metalloproteases analysis on the in vitro model of vein after intestinal passage. In (**A**) MMP-6 levels; in (**B**) MMP-9 levels. From (**A**) and (**B**) all the data are obtained with specific ELISA kits; * *p* < 0.05 vs. Control; α *p* < 0.05 vs. single agents; β *p* < 0.05 vs. Commercial product; γ *p* < 0.05 vs. KCl.

Finally, the last markers analysed were two MMPs (MMP-6 and MMP-9, Figure 5) as several studies have shown increased MMPs levels in patients with varicose veins. When compared to KCl, the individual agents led to a drop in MMP6 levels at all timings (*p* < 0.0001, Figure 5A), peaking at 24 h, when hypersmin and pumpkin seed extract levels were comparable to those of untreated cells (control). Like the previous analyses, the MIX and the commercial product induced a significant effect compared to KCl, the single agents and the control (*p* < 0.0001, about 62% more at 24 h vs. the commercial product). Like the Caspase 3 examination, the MIX consistently produces a more significant effect than the commercial product; however, the difference between the two becomes less apparent at 36 and 48 h, and the data are only important at 12 and 24 h (*p* < 0.001). However, there are some modest differences in the findings regarding MMP9 levels (Figure 5B). Indeed, in this case, hypersmin induced a significant decrease compared to KCl at all time frames (*p* < 0.001), while pumpkin seed was able to achieve that from 24 h (*p* < 0.001) and amaranthus starting from 36 h (*p* < 0.001). All the single agents induce a decrease in the levels of MMP9 over time. The commercial product restored MMP9 to levels slightly lower than the control one and significantly reduced levels compared to KCl and the single agents (*p* < 0.0001). Finally, the MIX induced better effects than all the other agents (*p* < 0.0001, about four times more at 24 h vs. the commercial product) with a peak at 4 h. These data show that the MIX of amaranthus extract, pumpkin seed extract and hypersmin can maintain the vein wall structure and function by regulating MMP levels.

## 4. Discussion

The entire range of venous diseases, including varicose vein development, are functionally referred to by the term CVI. Among the factors that can be identified as the causes of CVI are pregnancy, elevated body mass index and malfunctioning venous valves. These factors increase intraluminal pressure, venous hypertension and venous wall stress [44]. Generally, CVI is a complex condition requiring pharmacological, minimally invasive and conservative therapies [45]. Flavonoids, known for their venotonic, antioxidative and anti-inflammatory properties, are a key intervention. Studies have shown that flavonoids can modulate venous tone and reduce endothelial inflammation while exerting antioxidant effects, highlighting their therapeutic potential [13]. Indeed, by raising intracellular calcium levels, flavonoids have been shown to activate endothelial cells to produce NO [46] and have an antiangiogenic impact [47]. For instance, by inhibiting inflammatory mediators, diosmin protects microvascular permeability by influencing lymphatic drainage and microcirculation [48]. Indeed, a conventionally accepted therapy consists of a combination of diosmin and hesperidin, commonly called hypersmin. In this regard, this study studied the already known combination of diosmin and hesperidin but rendered it innovative by incorporating it with amaranthus and pumpkin seed extracts. Considering that this study aimed to create a novel nutraceutical formulation, firstly, we tested the absorption kinetics of the extracts selected on a gut-validated in vitro barrier model. The data obtained shows that all the chosen extracts can cross the intestinal barrier with a peak of absorption at 4 h. Additionally, the analysis of their combination demonstrated even more encouraging data, leading to the hypothesis that this novel formulation could be absorbed and thus be used as a treatment for CVI. To verify this hypothesis, the next step consisted of analysing if the MIX, after reaching the bloodstream, acts on dysregulated mechanisms in the CVI condition; indeed, the MIX increases cell viability, decreases oxidative stress levels and counteracts the inflammatory condition that arises during the pathophysiological processes of CVI. Chronic inflammation is known to cause structural damage to the venous wall, which aids in developing CVI. It is also recognised that as the condition progresses, the systemic indicators of inflammatory disease rise [49]. Thus, the data obtained on the MIX under investigation’s capacity to lower TNFα and IL-1β levels in contrast to the increase in the damage-mimicking CVI state is highly encouraging. Indeed, administration of the MIX for 48 h every 12 h shows the ability to consistently decrease the production of these inflammatory cytokines not only in comparison to the mimicked CVI condition but also to untreated cells, and especially significantly in comparison to a commercial product already present on the market. Therefore, these data provide the basis for using a novel MIX that outperforms currently available items. The data obtained on the inflammatory panel are aligned with the existing data in the literature about the anti-inflammatory potential of pumpkin seed [50], amaranthus [51], diosmin [18], and hesperidin [52].

Another mechanism involved in CVI pathophysiology is the activation of neutrophils that induces the increase in ROS levels [53]. However, this overproduction is also modulated by the action of the MIX; indeed, individual agents can reduce ROS production significantly compared to the CVI condition, but using the MIX substantially increases the effect. MIX composed of hypersmin, pumpkin and amaranthus also decreases ROS production compared to the control product, especially the commercial product. Again, this activity is consistent with the data already present in the literature on the antioxidant activity of all the single extract [18,50,51,52].

The endothelium controls vascular tone by releasing prostaglandins, hyperpolarising hormones and relaxing factors like NO. NO preserves the arterial wall, inhibits platelet adhesion and aggregation [54], and inhibits inflammation, thrombosis and cell proliferation in a healthy endothelium [55]. Physiologically, NO is produced by eNOS and has anti-inflammatory properties in the vein [56]; however, its production is deregulated in CVI condition. The MIX effectively regulates this mechanism, restoring NO and eNOS levels, surpassing the effectiveness of commercial products. Thus, this suggests that the MIX composed of hypersmin, pumpkin and amaranthus have a beneficial effect on controlling and preserving vascular tone. While diosmin and hesperidin’s ability to modulate NO and eNOS in endothelial cells is already known and well-established in the literature [57,58], there are less data on the ability of pumpkin seed and amaranthus to induce this effect.

These positive results were confirmed by the analysis of another marker affecting vascular tone, endothelin-1; in contrast to NO, endothelin-1 is a potent vasoconstrictor peptide [59]. Indeed, because endothelin-1 promotes inflammation, has mitogenic and proliferative effects and increases the production of free radicals and platelet activation, it has been linked to the development of vascular dysfunction [59]. As evidence of this, inducing the CVI state in vitro generates a significant increase in endothelin-1 levels. This increase, however, was strongly counteracted by the MIX for all timings examined, thus demonstrating its comprehensive ability to regulate vascular tone. Important findings emerge from this analysis, as the ability of pumpkin seeds and amaranth to modulate endothelin levels is still unknown.

The genesis and evolution of varicose veins are intimately tied to apoptotic problems in the vessel wall. Cell proliferation and apoptosis must be balanced for the environment to remain stable. As a result, structural alteration in the vein wall and apoptosis are closely associated [60]. The relationship between CVI and apoptosis has also been extensively studied. A study by Filis et al. showed that apoptotic deregulation is present in primary varicose veins; specifically, BAX and Caspase 3 expressions were increased in patients suffering from primary varicose veins compared with healthy patients [43]. Indeed, our investigation showed that apoptosis increased under CVI circumstances as levels of the apoptotic proteins BAX, Cyto-c, and Caspase 3 significantly rose after using KCl. Using the commercial product and the MIX reversed this state, with the latter having a significant effect. These findings demonstrate that the MIX can reduce apoptosis in addition to having anti-inflammatory and antioxidant properties and the capacity to modify vascular tone. While the data obtained from hypersmin are consistent with the literature [61,62], the data from the pumpkin and amaranth treatments revealed new and previously unexplored protective action of these two extracts.

CVI exhibits alterations in the amount of collagen and an imbalance in the protein components of the extracellular matrix; indeed, significant modifications in MMPs expression and activity are seen in varicose veins. Specifically, one important aspect that may contribute to increased MMPs expression and activity in varicose veins is elevated lower extremity venous hydrostatic pressure [63]. Once more, the MIX lessened this CVI-related factor. Indeed, the combination dramatically lowered MMP6 and MMP9 levels; again, this novel MIX outperformed the currently available product. These data confirm the data already present in the literature regarding the ability of hesperidin, diosmin and amaranthus to regulate MMP in different vascular diseases [64,65,66]; however, the data obtained from pumpkin seeds are quite novel, as so far this ability was not investigated in venous.

**Figure A1:**
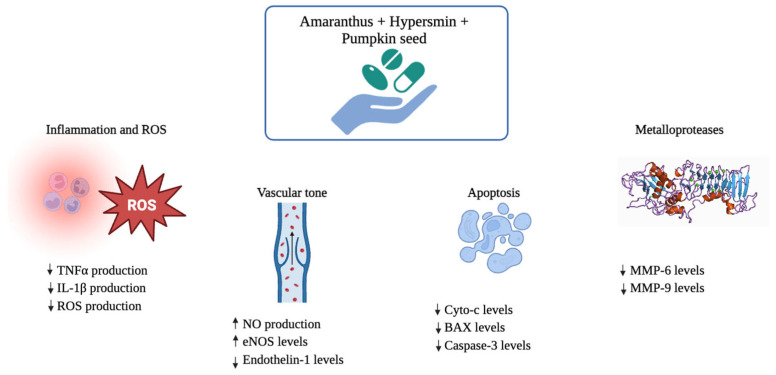
General schematic figure on the beneficial effects obtained with MIX in CVI condition.

The study’s findings suggest that this novel MIX of natural extracts can be absorbed and transported to the target, where it has a positive effect. In a CVI-induced setting, the combination of hypersmin, pumpkin and amaranthus is more effective than a commercial product in maintaining vascular tone and extracellular matrix activity, increasing NO production and slowing the apoptotic process (Figure A1 in Appendix A).

The results are encouraging; however, before concluding that this combination is effective, it is important to observe that some limitations are present due to the lack of verification from more complex models. To be precise, using this in vitro model, we did not study the entire metabolism of this combination and data on systemic circulation are also lacking. However, based on the positive data obtained and following a thorough investigation in more complex model, this nutraceutical combination might be considered for marketing. It is important to keep in mind that nutraceuticals do not readily fit into the legal classification of foods or medications, and there is currently no worldwide agreement on how to regulate them [67]. Because natural products are thought to be “low risk” and because it can be challenging to identify high-risk nutraceuticals that need biological and toxicological data, evidence of efficacy is frequently not needed [68]. However, given the promising results obtained from these preliminary analyses, the next step could be more in-depth analyses in animal models or through clinical trials to confirm the safety and efficacy of this new MIX before hypothesising to use this novel formulation for CVI treatment. Firstly, in vivo research or human clinical trials must validate these findings. Nonetheless, there is still reason to believe that oral administration of this novel MIX to humans could be a novel treatment approach to produce positive therapeutic benefits during CVI.

## 5. Conclusions

This study supports the hypothesis that this novel combination of natural extracts can be absorbed and delivered to the target. Further, the results of the current study on the effectiveness of a combination of hypersmin, pumpkin and amaranthus in humans may support the hypothesis that this can be a viable therapeutic strategy to achieve beneficial therapeutic effects in CVI; indeed, this combination was able to induce a reduction in different apoptotic markers and metalloprotease, while at the same time enhancing the maintenance of the vascular tone and demonstrating antioxidant and anti-inflammatory properties. However, the data require further validation, being this an in vitro study. Before assuming the possible efficacy of this combination, in vivo investigations or human clinical trials would be required to validate the clear and encouraging evidence obtained in vitro.

## Figures and Tables

**Table 1:** Characterisation of the extract studied.

Compound	Characterisation	Extract Solvent
Pumpkin seeds from *Curcubita maxima*	Alkaloids: 4.5–6.5% Flavonoids: 25.00–30.00 mg/g Phenolic compounds: 0.010–0.030 g/100 g Tannins: 0.005–0.020 g/100 g Saponins: 1.5–3.5 mg/g	Water
Amaranthus from *Amaranthus caudatus* L.	Stearic acid: 2–5% Palmitic acid: 18.5–21% Oleic acid: 25–35% Linoleic acid: 42–52% Squalene: 5–8% Total tocopherols: ≤1% Sterols: 1.0–2.5%	Ethanol and water
Hypersmin	Diosmin: ≥77% Hesperidin: ≥8% Acetoisovanillone: 0.09% Isorhoifolin: 1.81% Linarin: 1.94% Diosmetin: 0.42%	

## Data Availability

The Laboratory of Physiology stores raw data to ensure permanent retention under a secure system. This study’s data are available from the corresponding author upon reasonable request.

## Abbreviations

ATCC	American Type Culture Collection
CVI	Chronic venous insufficiency
DMEM-Adv	DMEM Advance
DMEM	Dulbecco’s Modified Eagle’s Medium
EGM-2	Endothelial Growth Medium-2
EMA	European Medicines Agency
eNOS	Endothelial nitric oxide synthase
FBS	Foetal bovine serum
FDA	Food and Drug Delivery
IL-1β	Interleukin 1β
MMP	Metalloproteases
NO	Nitric oxide
PAF	Platelet-activating factor
Papp	Apparent permeability coefficient
ROS	Reactive oxygen species
SOD	Superoxide dismutase
TEER	Trans-epithelial electrical resistance
TNFα	Tumour necrosis factor α
VEGF	Vascular endothelial growth factor

## References

[1] Orhurhu V., Chu R., Xie K., Kamanyi G.N., Salisu B., Salisu-Orhurhu M., Urits I., Kaye R.J., Hasoon J., Viswanath O. (2021). Management of lower extremity pain from chronic venous insufficiency: A comprehensive review. Cardiol. Ther..

[2] Bonkemeyer Millan S., Gan R., Townsend P.E. (2019). Venous Ulcers: Diagnosis and Treatment. Am. Fam. Physician.

[3] Kaplan R.M., Criqui M.H., Denenberg J.O., Bergan J., Fronek A. (2003). Quality of life in patients with chronic venous disease: San Diego population study. J. Vasc. Surg..

[4] Ortega M.A., Fraile-Martínez O., García-Montero C., Álvarez-Mon M.A., Chaowen C., Ruiz-Grande F., Pekarek L., Monserrat J., Asúnsolo A., García-Honduvilla N. (2021). Understanding Chronic Venous Disease: A Critical Overview of Its Pathophysiology and Medical Management. J. Clin. Med..

[5] Fukaya E., Flores A.M., Lindholm D., Gustafsson S., Zanetti D., Ingelsson E., Leeper N.J. (2018). Clinical and genetic determinants of varicose veins: Prospective, community-based study of ≈500,000 individuals. Circulation.

[6] Eberhardt R.T., Raffetto J.D. (2014). Chronic venous insufficiency. Circulation.

[7] Kahn S.R. (2016). The post-thrombotic syndrome. Hematol. Am. Soc. Hematol. Educ. Program..

[8] Raffetto J.D. (2018). Pathophysiology of Chronic Venous Disease and Venous Ulcers. Surg. Clin. N. Am..

[9] Mansilha A., Sousa J. (2018). Pathophysiological Mechanisms of Chronic Venous Disease and Implications for Venoactive Drug Therapy. Int. J. Mol. Sci..

[10] Ortega M.A., Romero B., Asúnsolo Á., Sola M., Álavrez-Rocha M.J., Sainz F., Álavrez-Mon M., Buján J., García-Honduvilla N. (2019). Patients with incompetent valves in chronic venous insufficiency show increased systematic lipid peroxidation and cellular oxidative stress markers. Oxid. Med. Cell. Longev..

[11] Kuhn J., Sultan D.L., Waqas B., Ellison T., Kwong J., Kim C., Hassan A., Rabbani P.S., Ceradini D.J. (2020). Nrf2-activating Therapy Accelerates Wound Healing in a Model of Cutaneous Chronic Venous Insufficiency. Plast. Reconstr. Surg..

[12] Horecka A., Biernacka J., Hordyjewska A., Dąbrowski W., Terlecki P., Zubilewicz T., Musik I., Kurzepa J. (2018). Antioxidative mechanism in the course of varicose veins. Phlebology.

[13] Casili G., Lanza M., Campolo M., Messina S., Scuderi S., Ardizzone A., Filippone A., Paterniti I., Cuzzocrea S., Esposito E. (2021). Therapeutic potential of flavonoids in the treatment of chronic venous insufficiency. Vasc. Pharmacol..

[14] Raffetto J.D., Khalil R.A. (2021). Mechanisms of lower extremity vein dysfunction in chronic venous disease and implications in management of varicose veins. Vessel. Plus.

[15] Perrin M., Ramelet A.A. (2011). Pharmacological treatment of primary chronic venous disease: Rationale, results and unanswered questions. Eur. J. Vasc. Endovasc. Surg..

[16] Imam F., Al-Harbi N.O., Al-Harbi M.M., Ansari M.A., Zoheir K.M.A., Iqbal M., Anwer M.K., Hoshani A.A.R., Attia S.M., Ahmad S.F. (2015). Pharmacological Research. Pharmacol. Res..

[17] Prasain J.K., Arabshahi A., Taub P.R., Sweeney S., Moore R., Sharer J.D., Barnes S. (2012). Simultaneous quantification of F2-isoprostanes and prostaglandins in human urine by liquid chromatography tandem-mass spectrometry. J. Chromatogr. B.

[18] Huwait E., Mobashir M. (2022). Potential and Therapeutic Roles of Diosmin in Human Diseases. Biomedicines.

[19] Lurie F., Branisteanu D.E. (2023). Improving Chronic Venous Disease Management with Micronised Purified Flavonoid Fraction: New Evidence from Clinical Trials to Real Life. Clin. Drug Investig..

[20] Lacatusu I., Arsenie L.V., Badea G., Popa O., Oprea O., Badea N. (2018). New cosmetic formulations with broad photoprotective and antioxidative activities designed by amaranth and pumpkin seed oils nanocarriers. Ind. Crops Prod..

[21] Lacatusu I., Badea G., Popescu M., Bordei N., Istrati D., Moldovan L., Seciuc A.M., Pantelid M.I., Rasitd I., Badea N. (2017). Marigold extract, azelaic acid and black caraway oil into lipid nanocarriers provides a strong anti-inflammatory effect in vivo. Ind. Crops Prod..

[22] Ionescu N., Neagu A.M., Raiciu A.D., Popescu M., Livadariu O. (2021). Characterization of some vegetable oils in order to use them in the treatment of the varicose veins. Rom. Biotechnol. Lett..

[23] Ruzmetova S., Abrorova M. (2022). Amaranth: Chemical Composition and Use Prospects. Sci. Innov..

[24] Gandhi S.V., Nilgar N.M., Bhalekar M.R. (2018). Formulation and evaluation of phytoconstituents cream for the treatment of varicose veins. World J. Pharm. Res. SJIF Impact Factor..

[25] Nardo A.E., Suárez S., Quiroga A.V., Añón M.C. (2020). Amaranth as a source of antihypertensive peptides. Front. Plant Sci..

[26] Smith A.B., Jones C.D., Nguyen E. (2015). Correlating in vitro and in vivo pharmacokinetics: Establishing dosage equivalence. J. Pharmacol. Sci..

[27] EMA Committee for Medicinal Products for Human Use (CHMP). https://www.ema.europa.eu/en/committees/committee-medicinal-products-human-use-chmp.

[28] Galla R., Grisenti P., Farghali M., Saccuman L., Ferraboschi P., Uberti F. (2021). Ovotransferrin Supplementation Improves the Iron Absorption: An In Vitro Gastro-Intestinal Model. Biomedicines.

[29] EMA International Council on Harmonisation of Technical Requirements for Registration of Pharmaceuticals for Human Use (ICH). https://www.ema.europa.eu/en/partners-networks/international-activities/multilateral-coalitions-initiatives/international-council-harmonisation-technical-requirements-registration-pharmaceuticals-human-use-ich.

[30] Uberti F., Lattuada D., Morsanuto V., Nava U., Bolis G., Vacca G., Squarzanti D.F., Cisari C., Molinari C. (2014). Vitamin D protects human endothelial cells from oxidative stress through the autophagic and survival pathways. J. Clin. Endocrinol. Metab..

[31] FDA M9 Biopharmaceutics Classification System-Based Biowaivers. https://www.fda.gov/media/117974/download.

[32] EMA ICH Guideline M9 on Biopharmaceutics Classification 5 System Based Biowaivers. https://www.ema.europa.eu/en/documents/scientific-guideline/ich-m9-biopharmaceutics-classification-system-based-biowaivers-step-2b-first-version_en.pdf.

[33] Galla R., Ruga S., Ferrari S., Saccone S., Saccuman L., Invernizzi M., Uberti F. (2022). In vitro analysis of the effects of plant-derived chondroitin sulfate from intestinal barrier to chondrocytes. J. Funct. Foods.

[34] Konishi Y., Hagiwara K., Shimizu M. (2002). Transepithelial transport of fluorescein in Caco-2 cell monolayers and use of such transport in in vitro evaluation of phenolic acid availability. Biosci. Biotechnol. Biochem..

[35] Molinari C., Morsanuto V., Ghirlanda S., Ruga S., Notte F., Gaetano L., Uberti F. (2019). Role of combined lipoic acid and vitamin D3 on astrocytes as a way to prevent brain ageing by induced oxidative stress and iron accumulation. Oxid. Med. Cell. Longev..

[36] Ruga S., Galla R., Ferrari S., Invernizzi M., Uberti F. (2023). Novel Approach to the Treatment of Neuropathic Pain Using a Combination with Palmitoylethanolamide and *Equisetum arvense* L. in an In Vitro Study. Int. J. Mol. Sci..

[37] Molinari C., Ruga S., Farghali M., Galla R., Bassiouny A., Uberti F. (2021). Preventing c2c12 muscular cells damage combining magnesium and potassium with vitamin D3 and curcumin. J. Tradit. Complement. Med..

[38] Lattuada D., Uberti F., Colciaghi B., Morsanuto V., Maldi E., Squarzanti D.F., Molinari C., Boldorini R., Bulfoni A., Colombo P. (2015). Fimbrial Cells Exposure to Catalytic Iron Mimics Carcinogenic Changes. Int. J. Gynecol. Cancer.

[39] Kaihara J.N.S., Minami C.K., Peraçoli M.T.S., Romão-Veiga M., Ribeiro-Vasques V.R., Peraçoli J.C., Palei A.C.T., Cavalli R.C., Nunes P.R., Luizon M.R. (2023). Plasma eNOS Concentration in Healthy Pregnancy and in Hypertensive Disorders of Pregnancy: Evidence of Reduced Concentrations in Pre-Eclampsia from Two Independent Studies. Diseases.

[40] Marey M.A., Yousef M.S., Liu J., Morita K., Sasaki M., Hayakawa H. (2016). Endothelin-1 downregulates sperm phagocytosis by neutrophils in vitro: A physiological implication in bovine oviduct immunity. J. Reprod. Dev..

[41] Partan R.U., Putra K.M., Kusuma N.F., Darma S., Reagan M., Muthia P., Radiandina A.S., Saleh M.I., Salim E.M. (2023). Umbilical Cord Mesenchymal Stem Cell Secretome Improves Clinical Outcomes and Changes Biomarkers in Knee Osteoarthritis. J. Clin. Med..

[42] Lee E.J., Zheng M., Craft C.M., Jeong S. (2021). Matrix metalloproteinase-9 (MMP-9) and tissue inhibitor of metalloproteinases 1 (TIMP-1) are localized in the nucleus of retinal Müller glial cells and modulated by cytokines and oxidative stress. PLoS ONE.

[43] Filis K., Kavantzas N., Isopoulos T., Antonakis P., Sigalas P., Vavouranakis E., Sigala F. (2011). Increased vein wall apoptosis in varicose vein disease is related to venous hypertension. Eur. J. Vasc. Endovasc. Surg..

[44] Pfisterer L., König G., Hecker M., Korff T. (2014). Pathogenesis of varicose veins—Lessons from biomechanics. Vasa.

[45] Miguel C.B., Andrade R.d.S., Mazurek L., Martins-de-Abreu M.C., Miguel-Neto J., Barbosa A.d.M., Silva G.P., Góes-Neto A., Soares S.d.C., Lazo-Chica J.E. (2025). Emerging Pharmacological Interventions for Chronic Venous Insufficiency: A Comprehensive Systematic Review and Meta-Analysis of Efficacy, Safety, and Therapeutic Advances. Pharmaceutics.

[46] Olszanecki R., Gebska A., Kozlovski V.I., Gryglewski R.J. (2002). Flavonoids and nitric oxide synthase. J. Physiol. Pharmacol..

[47] Wei Q., Zhang Y.-h. (2024). Flavonoids with Anti-Angiogenesis Function in Cancer. Molecules.

[48] Feldo M., Wójciak-Kosior M., Sowa I., Kocki J., Bogucki J., Zubilewicz T., Kęsik J., Bogucka-Kocka A. (2019). Effect of Diosmin Administration in Patients with Chronic Venous Disorders on Selected Factors Affecting Angiogenesis. Molecules.

[49] Nogueira J.F.L., Teixeira-Viana F.C., Barboza-Silva B.L., Mendes-Pinto D., Rodrigues-Machad M.D.G. (2023). Advanced Levels of Chronic Venous Insufficiency are Related to an Increased in Arterial Stiffness. Ann. Vasc. Surg..

[50] Hu Z., Hu C., Li Y., Jiang Q., Li Q., Fang C. (2024). Pumpkin seed oil: A comprehensive review of extraction methods, nutritional constituents, and health benefits. J. Sci. Food Agric..

[51] Ji H.S., Kim G., Ahn M.A., Chung J.W., Hyun T.K. (2022). Comparison of the antioxidant and anti-inflammatory activities of leaf extracts from grain amaranths (*Amaranthus* spp.). J. Plant Biotechnol..

[52] Buzdağlı Y., Eyipınar C.D., Kacı F.N., Tekin A. (2023). Effects of Hesperidin on Anti-Inflammatory and Antioxidant Response in Healthy People: A Meta-Analysis and Meta-Regression. Int. J. Environ. Health Res..

[53] Riaz B., Sohn S. (2023). Neutrophils in Inflammatory Diseases: Unraveling the Impact of Their Derived Molecules and Heterogeneity. Cells.

[54] Russo I., Barale C., Melchionda E., Penna C., Pagliaro P. (2023). Platelets and Cardioprotection: The Role of Nitric Oxide and Carbon Oxide. Int. J. Mol. Sci..

[55] Immanuel J., Yun S. (2023). Vascular Inflammatory Diseases and Endothelial Phenotypes. Cells.

[56] Apaijit K., Pakdeechote P., Maneesai P., Meephat S., Prasatthong P., Bunbupha S. (2022). Hesperidin alleviates vascular dysfunction and remodelling in high-fat/high-fructose diet-fed rats by modulating oxidative stress, inflammation, AdipoR1, and eNOS expression. Tissue Cell.

[57] Wójciak M., Feldo M., Borowski G., Kubrak T., Płachno B.J., Sowa I. (2022). Antioxidant Potential of Diosmin and Diosmetin against Oxidative Stress in Endothelial Cells. Molecules.

[58] Tarafdar A., Pula G. (2018). The Role of NADPH Oxidases and Oxidative Stress in Neurodegenerative Disorders. Int. J. Mol. Sci..

[59] Böhm F., Pernow J. (2007). The importance of endothelin-1 for vascular dysfunction in cardiovascular disease. Cardiovasc. Res..

[60] Gwozdzinski L., Bernasinska-Slomczewska J., Hikisz P., Wiktorowska-Owczarek A., Kowalczyk E., Pieniazek A. (2023). The Effect of Diosmin, Escin, and Bromelain on Human Endothelial Cells Derived from the Umbilical Vein and the Varicose Vein—A Preliminary Study. Biomedicines.

[61] Iriz E., Vural C., Ereren E., Poyraz A., Erer D., Oktar L., Gokgoz L., Halit V., Soncul H. (2008). Effects of calcium dobesilate and diosmin-hesperidin on apoptosis of venous wall in primary varicose veins. Vasa.

[62] Filis K., Kavantzas N., Dalainas I., Galyfos G., Karanikola E., Toutouzas K. (2014). Evaluation of apoptosis in varicose vein disease complicated by superficial vein thrombosis. Vasa.

[63] Chen Y., Peng W., Raffetto J.D., Khalil R.A. (2017). Matrix Metalloproteinases in Remodeling of Lower Extremity Veins and Chronic Venous Disease. Prog. Mol. Biol. Transl. Sci..

[64] Lee H.J., Im A.R., Kim S.M., Kang H.S., Lee J.D., Chae S. (2018). The flavonoid hesperidin exerts anti-photoaging effect by downregulating matrix metalloproteinase (mmp)-9 expression via mitogen activated protein kinase (mapk)-dependent signaling pathways. BMC Complement. Altern. Med..

[65] Feldo M., Wójciak-Kosior M., Sowa I., Kocki J., Bogucki J., Zubilewicz T., Bogucka-Kocka A. (2019). Monitoring of endostatin, TNF-a VEGFs, MMP-9, and cathepsin-L during three months of diosmin treatment in patients with chronic venous disease (CVD). Acta Angiol..

[66] Krishna P.S., Kumar N.R., Swathi, Rani S. (2023). Amaranthus viridis methanolic extract and its active compound kaempferol ameliorate myocardial infarction induced by isoproterenol through decreasing oxidative stress and cell death via Nrf-2/HO-1 and MMP/Bax/Bcl-2/TLR-4 pathways in rats. Comp. Clin. Pathol..

[67] Santini A., Novellino E. (2017). To Nutraceuticals and Back: Rethinking a Concept. Foods.

[68] Komala M.G., Ong S.G., Qadri M.U., Elshafie L.M., Pollock C.A., Saad S. (2023). Investigating the Regulatory Process, Safety, Efficacy and Product Transparency for Nutraceuticals in the USA, Europe and Australia. Foods.

